# Goat Milk Exhibits a Higher Degree of Protein Oxidation and Aggregation than Cow Milk During Cold Storage

**DOI:** 10.3390/foods14050852

**Published:** 2025-03-01

**Authors:** Lirong Zhu, Zixuan Fan, Wenhao Li, Yuanyuan Shan

**Affiliations:** 1 College of Food Science and Engineering, Northwest A&F University, Yangling 712100, China; 2 Shaanxi Union Research Center of University and Enterprise for Grain Processing Technologies, College of Food Science and Engineering, Northwest A&F University, Yangling 712100, China; 3 Shaanxi Engineering Research Centre of Dairy Products Quality, Safety and Health, Northwest A&F University, Yangling 712100, China

**Keywords:** goat milk, cow milk, protein oxidation, protein aggregation

## Abstract

Due to their markedly distinct protein compositions and structures, goat milk and cow milk display substantially different characteristics. In this study, the quality and composition of goat milk and cow milk were studied after being refrigerated at 4 °C for 7 days, with a particular focus on protein oxidation and aggregation states. The results revealed that alongside increases in acidity, microbial colony count, and hydrolysis, there was a significant change in the protein aggregation state beginning on the second day. This change was characterized by increased turbidity, an elevated centrifugal sedimentation rate, and a right-shifted particle size distribution. After seven days of refrigeration, the centrifugal sedimentation rate of goat milk increased from 0.53% to 0.97%, whereas that of cow milk rose from 0.41% to 0.58%. The degree of aggregation was significantly greater in goat milk compared to cow milk. Additionally, both protein and lipids exhibited substantial oxidation, with the degree of oxidation more pronounced in goat milk than in cow milk. The malondialdehyde (MDA) content increased from 0.047 μg/mL to 0.241 μg/mL in goat milk and from 0.058 μg/mL to 0.178 μg/mL in cow milk. The results suggest that goat milk was more prone to oxidation, which further reduced its stability. Therefore, in the storage and transportation of dairy products before processing, it is essential not only to monitor sanitary conditions but also to effectively control protein oxidation to enhance the quality of milk processing.

## 1. Introduction

Dairy products constitute a vital component of dietary nutrition which can be served as a valuable source of both macronutrients and micronutrients, and also contain many active compounds that play a crucial role in both nourishment and safeguarding health [1]. Cow milk constitutes a considerable share of the global market and is a vital source of protein in the human diet [2]. Goat milk is the third most consumed dairy product in the world, following cow milk and buffalo milk. In recent years, goat milk has gradually attracted widespread attention. Goat milk possesses unique characteristics [3], and these attributes make goat milk more suitable for infants, young children, the elderly, lactose-intolerant individuals, and other special groups [4].

Cow milk and goat milk exhibit different physicochemical and nutritional characteristics. For example, goat milk is characterized by higher levels of medium-chain fatty acids, ε-6 polyunsaturated fatty acids, ε-3 polyunsaturated fatty acids, and conjugated linoleic acid, as well as higher levels of calcium (Ca), phosphorus (P), magnesium (Mg), and copper (Cu) [4]. Additionally, goat milk has lower α_s1_-casein (α_s1_-CN) and higher β-casein (β-CN) and κ-casein (κ-CN) levels (these proteins are involved in hydrolysis and the cheese curdling process) [5]. Furthermore, in addition to differences in protein proportions, the size, hydration level, and mineralization of casein micelles in milk from different species exhibit variations [6]. The farming methods of dairy cows and dairy goats are also different. The industrial chain associated with dairy cows is more standardized, intensive, and refined. It is also well established, and milk is typically collected using an automated milking system, which enables the processing of cow milk from farm to factory in a short period via the cold chain, thereby ensuring the quality of the product. Dairy goat farming is an integral part of the national economy in many developing countries. The level of intensification in dairy goat farming remains relatively low, with the majority consisting of small-scale operations and dispersed feeding practices [7]. In some regions, extensive free-grazing management is still employed. Furthermore, scientific guidance is generally lacking, particularly for small farms where manual milking is commonly practiced. In addition, goat milk production is limited and subject to seasonal factors. The raw milk is typically collected and temporarily stored in milk storage tanks until it reaches a sufficient volume for processing [8,9,10]. This type of storage is not conducive to temperature control and affects the freshness and shelf life of raw materials. During this storage period, the protein, lipid, and lactose components constantly undergo complex biochemical reactions, including oxidation and hydrolysis catalyzed by microorganisms and enzymes [11]. These reactions result in a significant decline in milk quality [12]. For a long time, the proliferation of microorganisms and lipid oxidation have been considered the main causes of the decline in milk quality [9]. In recent years, protein oxidation has garnered growing attention [13].

Protein is a crucial ingredient of milk, determining its nourishment and processing performance. During the storage of raw materials, the lipolytic activity of lipase drives the yielding of free fatty acids, which are prone to oxidation under the action of endogenous substances, generating lipid radicals and reactive lipid oxidation products [14]. The protein backbone is affected by these reactive radicals [15], potentially resulting in changes to the protein’s structures and protein oxidation and aggregation [16]. Continuous protein oxidation leads to the formation of large aggregates through the establishment of covalent bonds (such as disulfide, dityrosine, and carbonyl amine reactions) or non-covalent interactions, leading to increasing turbidity [17] and alterations in particle size distribution [18]. Protein oxidation can lead to reduced nutritional value, flavor changes, and even harmful substances. Milk protein oxidation can take place at various stages throughout the dairy production process. For example, the heat treatment process can cause protein oxidation, leading to changes in protein digestibility [19]. Repeated freeze–thaw cycles could increase the level of oxidation of proteins and the proportion of irregular curls in the protein’s secondary structure, and could decrease its physical stability [4]. Low-temperature plasma could promote the oxidation and aggregation of casein and reduce the antioxidant capacity [20]. Bradley et al. (2006) showed that photo-induced oxidation is a key factor in accelerating milk deterioration [21]. The oxidation of histidine, tryptophan, and tyrosine affects the accessibility of rennet to casein [22,23], resulting in the loss of the ability of κ-CN and the entire casein to condense during curdling [24]. Additionally, the oxidation-induced aggregation of proteins may result in dairy products such as yogurt requiring more stringent control conditions, such as temperature and pH, during processing, thereby increasing production costs and complexity.

It has been confirmed that whey protein isolate can form heterogeneous aggregates primarily stabilized by protein–protein cross-linking, carbonyl cross-linking, and aromatic side chain cross-linking under oxidative stress [25]. However, the effects of oxidative modification on different processing properties of the protein are inconsistent. The issue of nutrient loss during processing is particularly prominent in milk, which is strongly linked to the raw milk’s quality, especially significantly influenced by factors such as the composition and structure of protein. Raw milk is susceptible to oxidative stress during refrigerated storage due to factors like enzymes, metal ions, and lipid oxidation products. Although some studies have explored the effects of processing conditions on protein oxidation and aggregation, there are relatively few studies on the properties associated with protein oxidation and aggregation in raw milk during refrigeration. There are differences in lipids, proteins, and minerals between goat milk and cow milk, which may lead to different changes in quality, protein oxidation, and aggregation during cold storage. For example, different protein species have different structures and amino acid compositions, which leads to varying sensitivities to oxidation [26]. An in-depth understanding of the protein changes in these two types of milk under cold storage conditions is of great significance for optimizing dairy processing technology, improving product quality, and extending shelf life. Therefore, this study investigated changes in the physicochemical properties and composition of cow milk and goat milk during cold storage at 4 °C for 7 days, with a particular focus on protein oxidation and aggregation states.

## 2. Materials and Methods

### 2.1. Materials

5,5′-dithiobis (2-nitrobenzoic acid) (DTNB) was purchased from Shanghai Aladdin Biochemical Technology Co., Ltd. (Shanghai, China). 1-anilino-8-naphthalene-sulfonate (ANS) and guanidine hydrochloride were purchased from Beijing Solarbio Science & Technology Co., Ltd. (Beijing, China). Trichloroacetic acid (TCA) was purchased from Shanghai Yien Chemistry Technology Co., Ltd. (Shanghai, China). 4,6-Dihydroxy-2-mercaptopyrimidine (TBA) was purchased from Shanghai Macklin Biochemical Co., Ltd. (Shanghai, China). All other chemicals were of analytical grade.

### 2.2. Sample Preparation

From October 2023 to December 2023, samples from five batches of raw milk were collected. Each batch of raw milk was obtained by mixing milk from 2 to 3 healthy animals (fed a uniform diet). To ensure a valid comparison between cow milk and goat milk, we collected raw milk from the same local farm (Yangling Shaanxi), where the cow milk was sourced from Holstein cows and the goat milk was sourced from Saanen dairy goats, and subjected the milk to identical storage and handling conditions. Each animal’s udder was cleaned with distilled water before manual milk collection. Raw milk was promptly placed in a container containing ice packs and delivered to the laboratory within 30 min. The raw goat milk and cow milk were individually distributed into polypropylene centrifuge tubes (10 mL each), sealed, and stored in a refrigerator at 4 °C. The milk samples were prepared in triplicate for each storage time and were analyzed immediately after being stored for various periods of 0, 1, 2, 3, 5, and 7 days, and each index was measured three times.

### 2.3. Determination of Centrifugal Sedimentation Rate

The centrifugal sedimentation rate was determined following the method described by Jensen et al. [27] with modifications. The sample (8 mL) was accurately pipetted into a centrifuge tube and centrifuged at 3500× *g* for 20 min. After this, the supernatant was decanted and the amount of sediment was measured (centrifugal sedimentation rate expressed in % *w*/*w*).

### 2.4. Determination of Turbidity

The protein sample (200 μL) was mixed with 4800 μL of distilled water, and the absorbance was measured at 633 nm (turbidity expressed in absorbance/protein concentration) [28].

### 2.5. Determination of Particle Size Distribution and ζ-Potential

The protein concentration of the sample was approximately adjusted to 0.5 mg/mL through dilution. Subsequently, a Nano laser particle size analyzer (ZEN3600, Malvern Instruments Ltd., Worcestershire, UK) was used to measure the ζ-potential and particle size distribution of milk protein at 25 °C.

### 2.6. Reducing and Non-Reducing Sodium Dodecyl Sulfate–Polyacrylamide Gel Electrophoresis

SDS-PAGE analysis was conducted according to Bao et al. [29] using a 5% polyacrylamide stacking gel and a 12% polyacrylamide resolving gel, and the thickness of the gel was 1 mm. Cow milk and goat milk were degreased (centrifuged at 3500× *g* for 20 min), and the upper lipid layer was removed. The protein samples were diluted with distilled water 20 times to achieve a protein concentration of approximately 1 mg/mL. The solution was then mixed with sample buffer (1:4 = *v*/*v*) containing β-Mercaptoethanol (β-ME) or without β-ME, followed by boiling for 10 min. Subsequently, each sample of 20 μL was loaded for electrophoretic analysis using an electrophoresis instrument (DYY-6C, Beijing Liuyi Biotechnology Co., Ltd., Beijing, China) in constant voltage mode. After electrophoresis, the protein bands in the gels were stained with Coomassie Brilliant Blue R-250 and decolorized using a solution of 10% acetic acid (*v*/*v*) and 5% ethanol (*v*/*v*). The gels were photographed with a gel imager (GE1DOC XR+, Bio-Rad Laboratories, Hercules, CA, USA).

### 2.7. Determination of Protein, Lipid, Lactose, Non-Fat Solid, and Total Solid Content

The samples were preheated at 37 °C for 20 min and then analyzed for protein, lipids, lactose, non-fat solids, and total solids using a dairy analyzer (FOSS FT1, Denmark FOSS Group Company, Hillerød, Denmark).

### 2.8. Determination of Acidity

The acidity was determined by titration using a standardized sodium hydroxide solution based on the method described by Manin et al. [30]. The sample (about 10.0 g) (*m*) was mixed with distilled water (20.0 mL) and a phenolphthalein indicator (2.0 mL) and then titrated with a standard solution of sodium hydroxide (0.10 M) (*c*). The volumes of sodium hydroxide standard solution consumed for the sample (*V*_1_) and water (*V*_0_) were recorded. Acidity was determined using the formula provided below.

Acidity = *c* × (*V*_1_ − *V*_0_) × 100/*m* × 0.1

### 2.9. Determination of pH

The pH of the sample was measured using a pH meter (PHSJ-4F, INESA Scientific Instrument Co. Ltd., Shanghai, China) according to the method reported in relevant studies [4].

### 2.10. Determination of Number of Aerobic Bacterial Colonies

The aerobic plate count was detected strictly according to the National Standards of the Republic of China [31]. The samples were diluted in sterile saline in a 10-fold gradient. Three appropriately diluted sample homogenates were selected for plating on plate count agar. After the agar solidified, the plates were incubated under controlled thermostatic conditions (37 °C) for a standardized period of 48 h. After incubation, only plates exhibiting colony counts within the range of 30 to 300 CFU, and with no evidence of spreading colonies, were selected for enumeration. The number of aerobic bacterial colonies was then calculated using the following formula:

$$N=\frac{\sum C}{(n_{1}+0.1n_{2})d}$$

where N represents the number of aerobic bacterial colonies, ∑C is the sum of the number of colonies in a plate (plate with a suitable range of colonies), n_1_ is the number of plates counted at first dilution, n_2_ is the number of plates counted at second dilution, and *d* is the dilution from which the first count was obtained.

### 2.11. Determination of Total Proteolytic Activity

Proteolytic activity was determined using azocasein as the substrate, following the protocol outlined by Zhang et al. [32] with a few modifications. Samples were centrifuged at 12,000× *g* for 10 min at 4 °C, followed by the mixing of 100 μL of the resulting supernatant with 500 μL of phosphate-buffered saline (PBS, 50 mM, pH 7.2). Subsequently, 100 μL of azocasein (1.5%, dissolved in 50 mM PBS) was added to the mixture. The combined solution was then incubated at 37 °C for 60 min, after which the reaction was terminated by adding 500 μL of trichloroacetic acid (20%). After centrifugation at 12,000 g/min for 5 min at 4 °C, the absorbance of the sample was measured at a wavelength of 366 nm. Proteolytic activity is expressed as the increase in absorption at 366 nm per hour and milliliter (∆A·h^−1^·mL^−1^).

### 2.12. Determination of Peroxide Value (POV)

The POV was determined using spectrophotometry according to the method described by Smet et al. [33], and the hydroperoxides were extracted with methanol and chloroform. First, 2 mL of milk was mixed with 2 mL of methanol. Subsequently, 4 mL of chloroform was added, and the mixture was vortexed for 30 s. The mixture was then centrifuged at 1500× *g* for 10 min, and then 1 ml of the underlying chloroform phase was transferred into the test tube and mixed with 0.98 mL of a chloroform/methanol (1:1) solution. Subsequently, 10 μL of potassium thiocyanate (30 g/L) and 10 μL of ferrous chloride (1.5 g/L) were added. The reaction mixture was incubated at room temperature for 5 min, after which the absorbance was measured at 500 nm. A standard curve was constructed using hydrogen peroxide solutions as the standard.

### 2.13. Determination of MDA Content

The determination of the MDA content of milk was performed according to the method of Zhao et al. [34] with some modifications. The sample (200 μL) was mixed with 5 mL TBA reaction combination solution (1.5% TBA + 15% TCA + 0.1% Disodium EDTA). The mixture was bathed in water at 100 °C for 30 min, and then taken out and cooled to room temperature. Next, 5 mL of the above-mentioned reaction solution was mixed with 5 mL of chloroform and subsequently centrifuged at 3000 r/min for 15 min. The absorbance at 532 nm was measured in the supernatant. The standard curve was constructed using 1,1,3,3-tetraethoxypropane as the standard.

### 2.14. Determination of Carbonyl Content

The carbonyl content in milk was determined by spectrophotometric analysis according to Bao et al. [29]. The outcomes are presented as the concentration of carbonyl groups (nmol/mg soluble protein) utilizing a molar extinction coefficient of 22,000 L/(mol·cm).

### 2.15. Determination of Dityrosine Content

The dityrosine content of protein was assessed using a modified version of the methodology proposed by Feng et al. [35]. The sample was diluted with phosphate-buffered saline (containing 0.6 M KCl, pH 6.0) to make a protein concentration of about 1 mg/mL. The dityrosine levels of samples were determined using a spectrophotometric method with an excitation wavelength of 325 nm and an emission wavelength of 420 nm (RF6000, Shimadzu, Kyoto, Japan). The content of dityrosine is expressed as absorbance per mg of protein.

### 2.16. Determination of Free Sulfhydryl Content

The free sulfhydryl content was determined according to the method of Bao et al. [29]. The sample (0.5 mL) was mixed with 2.0 mL of SDS solution (0.03 g/mL). Subsequently, 0.2 mL of DTNB solution (10 mM) was added, and the mixture was reacted in darkness for 1 h. The absorbance of the solution was then measured at 412 nm. The group without DTNB solution was used as the control group.

The number of free sulfhydryl groups is calculated as follows:

$$Free\;SH\;content=\frac{73.53\times D\times A_{412}}{C}$$

where A412 refers to the blank-subtracted absorbance value measured at a 412 nm wavelength, D indicates the experimental dilution ratio, and C is the protein concentration of the sample.

### 2.17. Determination of Surface Hydrophobicity

Surface hydrophobicity was determined according to the method of Wu et al. [36]. The sample was diluted to varying concentrations ranging from 0.01 to 1 g/L. Subsequently, the solution (4 mL) was mixed with ANS (50 μL, 8 mM) and placed in darkness for 2 min. The intensity of fluorescence was determined utilizing a fluorescence spectrometer (RF-6000, Shimadzu, Kyoto, Japan) at 365 nm (excitation wavelength) and 420 nm (emission wavelength). The relationship between the fluorescence intensity at 484 nm and the corresponding protein concentration (mg/mL) was plotted, and the linear slope calculated by linear regression analysis was the surface hydrophobicity of the protein.

### 2.18. Liquid Chromatography Tandem Mass Spectrometry (LC/MS)

The goat milk samples were randomly divided into two groups. One group was fresh milk, and the other group was milk that had been stored for 2 days. All samples were centrifuged at 12,000× *g* for 20 min at 4 °C, and the top fat layer was removed to collect skim milk. The Bradford protein assay was used to determine protein concentration. Protein samples were processed through the following workflow: Initially, aliquots were adjusted to a final volume of 100 μL using DB dissolution buffer (8 M urea, 100 mM triethylammonium bicarbonate (TEAB), pH 8.5). Sequential enzymatic digestion was performed by first adding trypsin in 100 mM TEAB buffer, followed by vortex mixing and incubation at 37 °C for 4 h. Subsequently, a secondary digestion phase was initiated with additional trypsin and CaCl_2_, extending the reaction at 37 °C for 12 h. The resulting peptides were acidified with formic acid to achieve a pH under 3.0 and then clarified by centrifugation (12,000× *g*, 5 min, 25 °C). The supernatant was slowly loaded into the C18 desalting column with the following sequence: (1) three washes with equilibration solution (3% acetonitrile/0.1% formic acid (FA)) and (2) elution with 70% acetonitrile/0.1% FA. Eluted fractions were pooled, lyophilized, and reconstituted in 100 μL 0.1 M TEAB. TMT labeling reagents (41 μL in anhydrous acetonitrile) were incubated at room temperature for 2 h with continuous agitation. Reactions were quenched with 8% ammonia. All labeling samples were mixed with equal volume, desalted, and lyophilized.

Mobile phase A (100% water, 0.1% formic acid) and liquid B (80% acetonitrile, 0.1% formic acid) were prepared for LC. A total of 1 μg of each fraction as the sample was injected into the LC-MS/MS system for detection. The EASY-nLC^TM^ 1200 nano-upgraded UHPLC sample system was used. The precolumn (4.5 cm × 75 μm, 3 μm) and analytical column (25 cm × 150 μm, 1.9 μm) used in this project were both home-made. The gradient elution settings were as follows: 0–2 min, 6–15% B; 2–48 min, 15–40% B; 48–50 min, 40–50% B; 50–51 min, 50–55% B; 51–60 min, 55–100%.

Liquid chromatography-separated peptides were subjected to high-resolution mass spectrometric analysis using a Q Exactive ^TM^ HF-X mass spectrometer (Thermo Fisher Scientific, Waltham, MA, USA) configured with a Nanospray Flex™ (ESI) (Thermo Fisher Scientific, Waltham, MA, USA) ion source. Instrumental settings included the following: electrospray ionization voltage maintained at 2.1 kV and an ion transport capillary temperature of 320 °C. Full-scan spectra were acquired over *m*/*z* 350–1500 with 60,000 resolving power (defined at *m*/*z* 200), an automatic gain control (AGC) target value of 3 × 10^6^, and a maximum ion injection time of 20 ms. Data-dependent acquisition (DDA) mode selected the 40 most intense precursors for HCD fragmentation (normalized collision energy: 32%), with MS/MS spectra recorded at 30,000 resolution (*m*/*z* 200). Secondary mass analysis parameters comprised the following: 5 × 10^4^ AGC targets, 54 ms injection duration, intensity threshold of 1.2 × 10^5^ counts, and 20 s dynamic exclusion window.

Mass spectrometry data analysis was performed using Proteome Discoverer software 3.2 (Thermo Fisher Scientific, Waltham, MA, USA) with independent database searches conducted for each experimental run against the UniProt protein database. The search parameters were configured with the following specifications: precursor ion mass accuracy was maintained at ±10 ppm, while fragment ion mass tolerance was set to 20 millidaltons.

### 2.19. Statistical Analysis

The experimental data were subjected to analysis of variance (ANOVA) at a significance level of *p* < 0.05 using the statistical package SPSS 26.0 (SPSS Inc., Chicago, IL, USA). The results are presented as the mean ± standard deviation (SD) of three independent experiments. Origin 2021 (Origin2018, OriginLab Corporation, Northampton, MA, USA) was used to plot and display the data.

## 3. Results

### 3.1. Protein Aggregation of Raw Goat Milk and Cow Milk During Cold Storage

**Figure 1:**
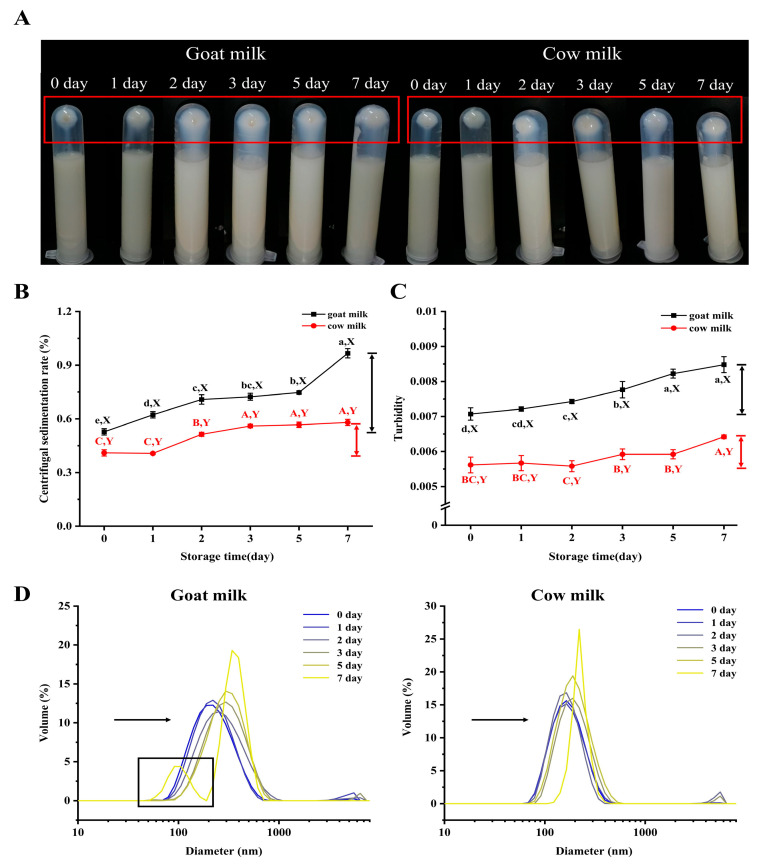
Changes in centrifugal precipitation (**A**), centrifugal sedimentation rate (**B**), turbidity (**C**), and particle size distribution (**D**) of raw milk during refrigeration. Different letters (a–c and A–C) indicate significant differences (*p* < 0.05) between samples (lowercase letters for goat milk; capital letters for cow milk). Different letters (X, Y) indicate significant differences (*p* < 0.05) between samples of different types of milk stored for the same period.

The aggregation behavior of milk was examined in this study under cold storage conditions. As shown in Figure 1B, after 7 days of refrigeration, the centrifugal sedimentation rate of goat milk increased from 0.53% to 0.97%, while that of cow milk rose from 0.41% to 0.58%, and goat milk exhibited a higher degree of protein precipitation compared to cow milk (Figure 1A). Furthermore, the turbidity of goat milk increased from 0.0071 to 0.0085, while that of cow milk rose from 0.0056 to 0.0064. The initial turbidity measured in goat milk was higher than in cow milk, implying that the protein particles in goat milk are larger compared to those found in cow milk. These findings align with previous research by Yang et al. [37], who showed that the average particle diameter of cow milk is 161.0 nm and that of goat milk is 209.8 nm. The results of turbidity and the centrifugal sedimentation rate indicated that goat milk exhibited a greater extent of protein aggregation compared to cow milk during cold storage. Figure 1D shows the results of particle size distribution, which reveal a gradual shift toward larger particles in both goat and cow milk during refrigeration. Especially from the second day, a noticeable change in particle distribution occurred. After 7 days of refrigeration, a slight peak was noted in the particle size distribution of goat milk. This indicates that the protein was hydrolyzed. Milk protein is a critical component that influences the physicochemical characteristics, microstructure, sensory attributes, and nutrients of dairy products. In liquid milk, protein aggregation can lead to precipitation, thereby compromising sensory quality and reducing shelf life. In yogurt, protein aggregation results in decreased water retention and a grainy texture, adversely affecting taste and smoothness. Pulsed light treatment promotes the rearrangement of protein structures by exposing tryptophan and tyrosine residues and increasing disulfide (S-S) bonds through oxidation, which subsequently induces protein aggregation. This process causes ricotta cheese particles to fragment into smaller units that interact further and reassemble into larger aggregates, thereby altering the product’s texture and potentially generating an unpleasant odor (Ricciardi et al., 2021 [38]). Many factors can induce protein aggregation, such as pH, oxidation, and enzymes, among others [39], and these will be analyzed later.

**Figure 2:**
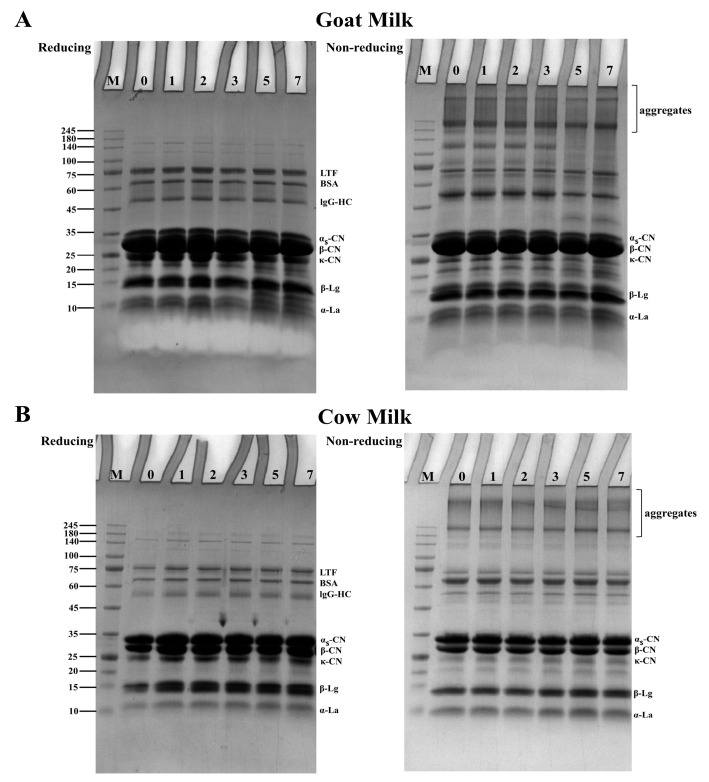
SDS-PAGE of goat milk (**A**) and cow milk (**B**) during cold storage. The letter (M) indicates standard protein markers, and the numbers 0, 1, 2, 3, 5, and 7 indicate the number of cold storage days.

SDS-PAGE was conducted to observe possible changes in protein patterns during cold storage. As shown in Figure 2, the results are consistent with previous research [5]. Under non-reducing electrophoresis conditions, protein aggregations (>140 kDa) were observed in both goat milk and cow milk. Under reducing conditions, certain bands disappeared while the intensity of casein and whey protein bands increased, indicating that disulfide bonds were involved in protein cross-linking and aggregation. There was no obvious difference in the bands for goat milk and cow milk when the storage time of milk was 2 days, which aligned with the observed changes in particle size distribution. New protein bands were observed as the storage time of milk increased to 5–7 days, and the molecular weights of these ranged between 10 and 25 kDa. The results showed that protein was hydrolyzed after 5 days of refrigeration. Higher levels of hydrolysis were observed in goat milk, possibly attributed to its protein structure which facilitates more efficient enzymatic activity. Previous studies have also shown that the proteins in goat milk undergo hydrolysis at a faster and more extensive rate compared to those in cow milk [37]. The enzymes that cause proteolysis include endogenous enzymes as well as those produced by microorganisms. These enzymes could result in the hydrolysis of κ-CN and β-CN, etc., thereby damaging the quality of milk products [40]. Both goat milk and cow milk primarily include casein (α_s_-CN, β-CN, and κ-CN) and whey protein (α-La and β-LG). As shown in Figure 2, the casein of goat milk contained a larger proportion of β-CN and a smaller proportion of α_s_-CN than cow milk. α-CN contains numerous phosphorylated groups that can bind to colloidal calcium phosphate (CCP), thereby stabilizing the micellar structure. β-CN is maintained within the micelle through ionic bonds, hydrophobic interactions, and the partial binding of phosphate groups to CCP [41]. The phosphorylated groups confer a negative charge to the micelles, which helps prevent micelle aggregation. Due to the different phosphorylation degrees of different caseins, micelles composed of different proportions of casein have different hydration, size, and sensitivity to the environment. Goat milk’s casein micelles are larger in particle size than those of cow milk [42], contain more calcium and inorganic phosphorus, and have lower solubility, and the higher calcium ion activity of κ-CN makes it easier to dissociate from the micelle surface, so the proteins in goat milk are less stable [43]. Certain bands of hydrolysates (between 10 and 15 kDa) existed in the reduced state but disappeared under non-reducing conditions from the bands on days 5 and 7 of refrigeration, which indicated that protein hydrolysates could participate in aggregate formation through disulfide bonds. Some hydrolysates of protein could also recombine to form aggregates under the action of calcium or through other interactions [44]. Previous studies have also demonstrated that the degree of proteolysis did not increase significantly after 3 days of storage at 6 °C. The degree of proteolysis increased from 6.7% to approximately 12% after 7 days of storage [45], and β-CN and κ-CN were dissociated first into the whey phase [46]. However, the hydrolysis of κ-CN notably impacted the stability of casein micelles [47].

### 3.2. Changes in Major Components and Quality of Raw Goat and Cow Milk During Cold Storage

This study highlighted that goat milk exhibited more pronounced aggregation compared to cow milk during the same period, suggesting that goat milk protein is more sensitive to environmental changes. Aggregation behavior is significantly influenced by the chemical composition and physicochemical properties of raw materials. As shown in Table 1, the results of chemical composition analysis showed that fresh goat milk contained higher protein levels but a lower fat content, with similar lactose levels. The observed lactose levels in this study were slightly lower than conventional values. This variation can be attributed to multiple interacting factors, such as breed characteristics, physiological status, dietary composition, and seasonal variations. A diet containing more hay may be one of the reasons for low lactose levels in this study (a low-calorie-density diet has been shown to significantly reduce lactose levels in milk) [48,49]. The total amount of protein did not change significantly, especially in the first two days, and the SDS-PAGE results did not change significantly (therefore, the types of and changes in milk proteins were analyzed by proteomics later). This is consistent with the results of Zhuang et al. [50]. However, the content of lipids and lactose decreased during later periods of storage, which could be linked to fatty hydrolysis and microbial proliferation. Microorganisms and enzymes act on these organic substances to make them oxidize and hydrolyze, leading to alterations in their physical and chemical characteristics [51]. Therefore, the pH, acidity, total colony count, and protease activity during cold storage were determined.

As shown in Table 2, the titrated acidity of both goat milk and cow milk increased significantly (*p* < 0.05) during cold storage, while pH exhibited a decreasing trend. During cold storage, the pH of goat milk decreased from 6.701 to 6.411, while the pH of cow milk decreased from 6.801 to 6.393. The decrease in pH mainly occurred in the later stage of cold storage. Goat milk exhibited a lower initial pH compared to cow milk. This is in agreement with Tan et al. [52] who also observed that goat milk had a lower pH (6.34) compared to cow milk (6.59). This reduction in pH was related to the activity of microorganisms (mainly psychrophilic bacteria) and the dissociation of micellar calcium [51], which could lead to a decrease in electrostatic repulsion and a shrinking of the surface hairy layer of micelles [53]. Hence, both steric hindrance and electrostatic repulsion were compromised and eventually increased aggregation [42,54]. The protein aggregation of goat milk was higher. However, the pH of cow milk changed more than that of goat milk after 7 days, indicating that pH alone might not be the main factor affecting the aggregation differences between the two types of milk. The results indicated that pH had a great influence on protein aggregation in the late refrigeration period. Acidification between pH 6.7 and 6.0 could not induce casein micelle dissociation [55]. Therefore, the hydrolysis of protein in the later period of cold storage was mainly caused by the action of microorganisms and enzymes. Total colony counts exceeded 1 million on the third day in the two types of milk. The cold chain is essential for minimizing the growth of microorganisms and ensuring the quality of raw materials. Nevertheless, the proliferation of psychrotrophic bacteria (such as *Pseudomonas*) during refrigeration can lead to the production of heat-stable enzymes that may deteriorate milk’s sensory quality and other dairy products [40]. Therefore, raw milk should not be refrigerated for extended periods, particularly since pasteurized milk has stricter raw material requirements. There are notable differences in the microbial quality standards for raw materials used in pasteurized milk across various countries and regions. For instance, some enterprises in China stipulate that the total bacterial count should not exceed 100,000 CFU/ mL and the duration of storage for raw materials should be limited to no more than 12 h. Stratakos et al. demonstrated that pasteurization resulted in a 1.19 log CFU/mL reduction in the total viable bacteria count and significantly decreased the numbers of *Enterobacteriaceae*, lactic acid bacteria (LAB), and *Pseudomonas* [56]. However, throughout most of the storage period, no significant (*p* < 0.05) difference was observed in either total aerobic bacterial colony counts or protease activity between goat milk and cow milk, indicating that microbial load and enzyme activity were not the main reasons for the difference in protein aggregation between the two milk types.

Many factors make the protein of goat milk more sensitive to environmental changes, such as the smaller size of casein molecules [53], lower α_s_-CN content, higher concentration of free calcium ions in the whey phase, and higher calcium ion activity of κ-CN, making it easier to dissociate from the micelle surface [57]. In addition, the natural oxidative stress environment in raw milk could also influence protein structure during storage and transportation, potentially triggering protein aggregation [58]. Therefore, we compared the differences in protein and lipid oxidation between goat milk and cow milk during refrigeration from the perspective of the oxidation-induced formation of aggregates.

### 3.3. Changes in Lipid and Protein Oxidation of Raw Goat Milk and Cow Milk During Refrigeration

**Figure 3:**
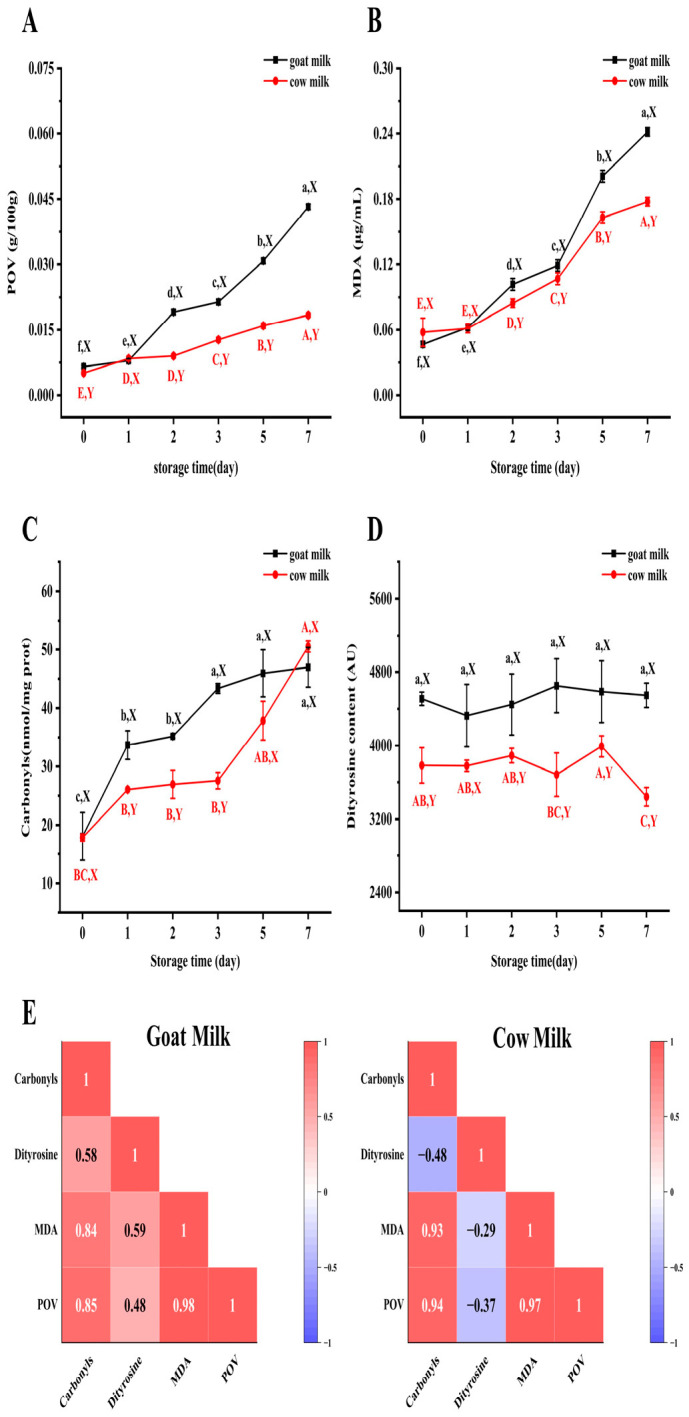
POV (**A**), MDA (**B**), carbonyl (**C**), dityrosine (**D**), and correlation analysis (**E**) of raw goat milk and cow milk during cold storage. Different letters (a–f and A–E) indicate significant differences (*p* < 0.05) between samples (lowercase letters for goat milk; capital letters for cow milk). Different letters (X, Y) indicate significant differences (*p* < 0.05) between samples of different types of milk stored for the same period.

The POV and MDA reflect the levels of primary and secondary metabolites of fat oxidation, respectively, serving as indicators of the extent of fat oxidation [59]. As shown in Figure 3, the POV and MDA content increased significantly (*p* < 0.05) during cold storage. On the second day of refrigeration, the POV in goat milk and cow milk increased by 172.86% and 77.00%, respectively, and the MDA content increased by 118.52% and 50.82%, respectively, which demonstrated increased lipid oxidation. Milk is exposed to oxidative stress during storage. Over time, the antioxidant capacity of the milk system decreases, leading to lipid oxidation due to the influence of metal ions, temperature fluctuations, and oxidases. Furthermore, the POV and MDA levels in goat milk were notably elevated (*p* < 0.05) compared to those in cow milk following the second day, indicating that the fat oxidation degree of goat milk was higher. The same conclusion was reached in the study of Oancea et al. [60], where the MDA content of goat milk increased from 161.25 μg/L to 182.9 μg/L, while that of cow milk increased from 97.37 μg/L to 105.2 μg/L, after storage for 24 h at 20 °C. This result was related to the fact that goat milk contains more polyunsaturated fatty acids and has a smaller diameter of fat globules which could provide a larger functional surface area and make them more susceptible to oxidation reactions [61]. Fat oxidation can impede food quality by adversely affecting flavor, color, texture, and structure [62]. Substances like free radicals and aldehydes resulting from fat oxidation can also induce protein oxidation and aggregation [63]. In addition, both primary products (such as hydroperoxides) and secondary products (such as aldehydes) produced by lipid oxidation can react with proteins, thereby inducing protein aggregation. Free radicals produced during lipid oxidation can extract hydrogen atoms from protein peptide chains and amino acid side chains to generate oxidation products, which can undergo secondary reactions with amine nucleophilic groups (such as Lys residues), leading to protein cross-linking [26]. Aldehydes, the secondary oxidation products of fatty acids, are electrophilic and can react with nucleophilic groups in proteins, and form complexes with the nitrogen or sulfur centers of active amino acid residues through electrostatic and hydrophobic attraction [64]. Whey protein isolate (WPI) exhibited sensitivity to the lipid oxidation product MDA, and the covalent interaction between MDA and WPI significantly influences the physicochemical properties of WPI [65]. Some measures can be taken to reduce oxidation, such as maintaining a low-temperature and -light environment, reducing vibration and impact during transportation and adding antioxidants.

To evaluate the level of protein oxidation, the contents of dityrosine and carbonyl groups were determined during cold storage. As shown in Figure 3C, the carbonyl content increased significantly (*p* < 0.05) during cold storage. The levels of carbonyl groups in both goat and cow milk increased by 95.06% and 50.87%, respectively, when the storage time of milk was 2 days. Meanwhile, during the initial five days of refrigeration, the carbonyl content in goat milk consistently exceeded the level in cow milk. One of the reasons for this difference may be the elevated fat oxidation in goat milk, which leads to the generation of more free radicals and active substances that promote protein oxidation. In addition, different amino acids have varying sensitivities to oxidation [26]. Liu et al. [66] conducted research demonstrating that methionine ranked among the amino acid residues exhibiting the highest sensitivity to oxidation. The results of Lamothe et al. indicate that the different antioxidant capacities of whey protein and casein protein were related to their amino acid compositions and structures, and that the ability of the two proteins to chelate transition metal ions and clear free radicals was different [67]. Therefore, different protein compositions may lead to variations in oxidative sensitivity between goat milk and cow milk. However, specific differences in oxidative sensitivity between goat milk and cow milk proteins require further study. Correlation analysis revealed a strong relationship (r > 0.8) between carbonyl content and both the POV and the MDA levels. However, this correlation remains observational, as confounding factors inherent in the milk system (e.g., metal ions, vitamins) may affect the oxidation process. Although previous studies have demonstrated that lipid oxidation and protein oxidation can promote one another in a simple model [13], given the complexity of the milk system, further exploration is needed. Dityrosine is an important product of protein oxidation [68]. However, it did not show a significant (*p* < 0.05) increase during refrigeration, which was probably caused by a change in protein structure. Protein oxidation can affect the nourishment, processing, and functional characteristics of dairy products. The oxidized flavor compounds produced by photo-oxidation are among the most common odors in liquid milk and seriously affect the sensory quality of the product [69]. In addition, protein oxidation could reduce the sensitivity of isolated whey proteins to proteases, and the physicochemical and structural changes caused by high oxidation (such as protein cross-linking) could mask the site of enzyme cleavage, thereby reducing its digestibility [70]. At the same time, harmful substances are produced during the oxidation process, and dietary consumption of oxidatively modified milk proteins can induce oxidative stress in murine models, resulting in deficiencies in their spatial navigation and memory abilities [71]. Oxidation can affect protein solubility and other properties, thus impacting gelation and emulsification functions, adversely impacting the stability and quality of food and limiting the applications of dairy protein in food [13]. A greater degree of oxidation of goat milk’s proteins would have an important effect on processing; for example, it would affect the control of the texture of cheese [72], make proteins more likely to precipitate during heat treatment [73], and influence the curdling process [74]. Protein oxidation not only leads to reduced edible quality [75] but also causes protein aggregation through covalent bonds or non-covalent forces [76]. Free radicals have the potential to attack the amino acid residues and protein backbone chains, leading to structural modifications and altering the chemical forces that maintain protein structure [77]. Research by Shang et al. [78] demonstrated that lipid oxidation caused by increased pork back contents of grass carp surimi resulted in protein oxidation and structural changes. Therefore, the higher level of protein oxidation in goat milk may contribute to its higher protein aggregation compared to cow milk. However, this study lacked long-term storage experiments and an examination of the effect of environmental factors (e.g., temperature) on milk protein oxidation and aggregation, which need to be further explored.

### 3.4. Changes in Surface Properties of Raw Goat and Cow Milk During Cold Storage

**Figure 4:**
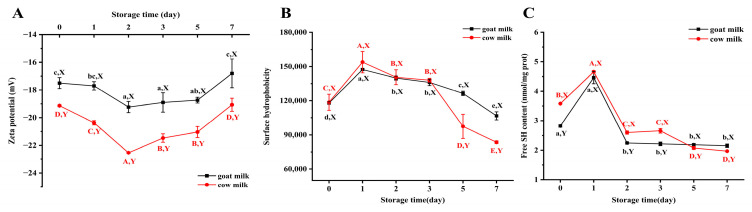
Changes in ζ-potential (**A**), hydrophobicity (**B**), and free sulfhydryl (**C**) content of raw goat milk and cow milk during cold storage. Different letters (a–d and A–D) indicate significant differences (*p* < 0.05) between samples (lowercase letters for goat milk; capital letters for cow milk). Different letters (X, Y) indicate significant differences (*p* < 0.05) between samples of different types of milk stored for the same period.

As shown in Figure 4A, the ζ-potential of goat and cow milk reached a minimum on the second day of refrigeration. However, the ζ-potential of both types of milk displayed no significant (*p* < 0.05) change after storage at 4 °C for 7 days compared to at the beginning, which is consistent with the observations reported by Liu et al. [55]. Interestingly, the ζ-potential of goat milk remained elevated compared to that of cow milk throughout the storage period. This was the result of the combined action of pH reduction and protein hydrolysis (goat milk protein exhibited a higher degree of hydrolysis, resulting in the shedding of charged peptides) [79,80]. Meanwhile, free fatty acids from lipid hydrolysis could also bind to the micelles, resulting in an increase in ζ-potential, which could be offset by the decrease in ζ-potential caused by the dissociation of protein peptides [81]. Thus, the ζ-potential on the protein surface was actually higher than what was measured.

The structural integrity of casein micelles is primarily maintained by the delicate equilibrium of hydrophobic interactions and electrostatic interactions [82]. Notably, the levels of free sulfhydryl groups and hydrophobicity exhibited an initial increase on the first day, followed by a continuous decline, which indicated that hydrophobic interactions and disulfide bonds played a role in the formation of protein aggregates [83]. No significant alterations were observed in the pH levels during the early stage of refrigeration, but significant oxidation of proteins and lipids occurred, which indicated that protein changes were predominantly induced by oxidation during the pre-cold storage period. The mild oxidation of proteins in the presence of an oxidant (such as free radicals and lipid oxidation products) leads to the denaturation, dissociation, and peptide chain expansion of protein subunits, thereby exposing sulfhydryl groups and hydrophobic amino acid side chains (including both aliphatic and aromatic residues) to a polar environment [84]. As the degree of protein oxidation increases, free radicals attack protein residues, leading to amino acid radical polymerization. The exposed hydrophobic amino acid residues and sulfhydryl groups can also induce protein aggregation through hydrophobic interactions and the formation of disulfide bonds. As the cold storage period progressed, sustained protein oxidation, the pH decrease, and the action of enzymes collectively destroyed the protein structure, intensifying hydrophobic interaction and fostering the formation of intramolecular and intermolecular disulfide bonds [85], which resulted in diminished protein stability and the formation of a substantial amount of aggregates [86].

### 3.5. Changes in Main Proteins of Goat Milk During Cold Storage

**Figure 5:**
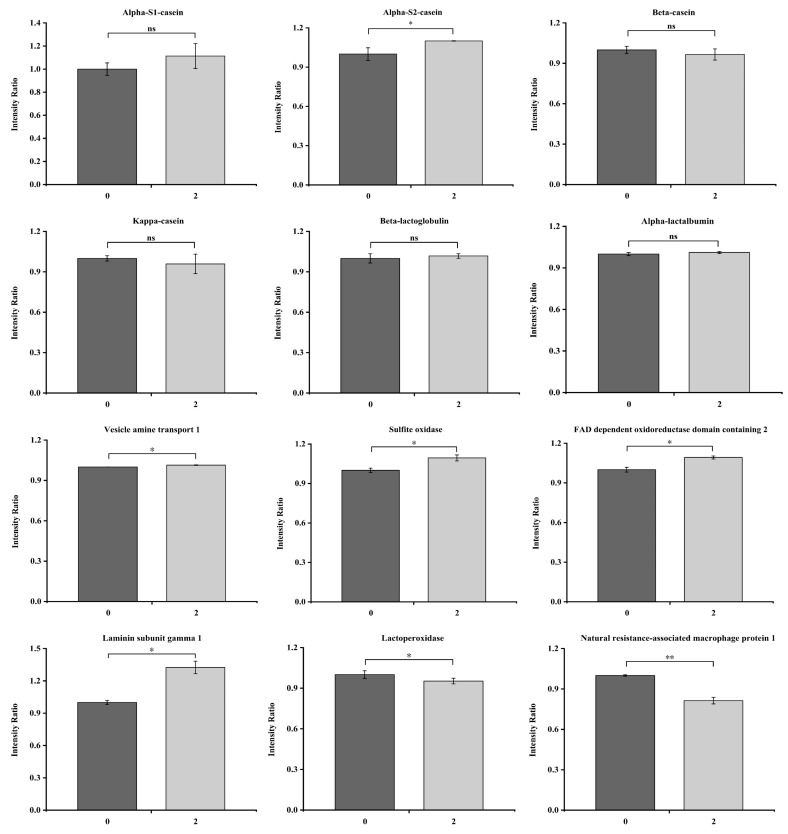
Changes in main proteins and certain enzymes of raw goat milk on the 0th and 2nd day of cold storage. “ns” means that there is no significant difference between samples (*p* < 0.05); “*” means that there is a significant difference between samples (*p* < 0.05); “**” means that there is a significant difference between samples (*p* < 0.01).

The results described above indicate that goat milk exhibited a greater level of aggregation than cow milk, and the second day was the key period for milk quality and aggregation. Therefore, changes in the main proteins of goat milk that was stored for 0 and 2 days were evaluated. As shown in Figure 5, except for α_s2_-CN, no significant (*p* < 0.05) changes were noted in the proteins, indicating that the composition of goat milk protein changed little during the first 2 days of refrigeration. These findings further support the conclusion that protein oxidation primarily drove changes in protein structure during the early cold storage stage. In addition, a notable rise (*p* < 0.05) in *Laminin subunit gamma 1* concentrations was observed after 2 days of refrigeration, which is a protein complex formed through the association and bonding of multiple proteins in specific arrangements, indicating that protein began to aggregate.

Based on the above results demonstrating the higher oxidation of fat and protein in goat milk, the changes in specific proteins associated with oxidation were examined on days 0 and 2 of refrigeration. Lactoperoxidase (LPO), a natural heme-containing oxidoreductase enzyme found in milk, collaborates with thiocyanate ion (SCN) and hydrogen peroxide (H_2_O_2_) to generate hypothiocyanite ion (OSCN), which possesses wide-ranging antimicrobial effects against pathogenic organisms [87]. Natural resistance-associated macrophage protein 1 (NRAMP1) is a cofactor for catalase and superoxide dismutase (SOD), essential antioxidant enzymes responsible for combating oxidizing agents like free radicals [88,89]. There was a notable reduction in the levels of LPO and NRAMP1 after two days of refrigeration, indicating that the antioxidant capacity of the milk system was decreased. Sulfite oxidase (SO) is involved in the final stage of the oxidative degradation of sulfur-containing amino acids, catalyzing the conversion of sulfites to sulfates, including methionine, cysteine, and glutathione [90]. Vesicle amine transport 1 (VAT-1) is a membrane protein within the NAD(P)-dependent quinone oxidoreductase subfamily, which catalyzes REDOX reactions [91]. Fad-dependent oxidoreductase domain-containing 2 (FOXRED 2) can interact with Flavin adenine dinucleotide (FAD), a coenzyme essential for various flavoprotein oxidoreductases, participating in reactions like fatty acid oxidation and energy metabolism [92]. This enzyme also facilitates the incorporation of a single oxygen atom from molecular oxygen into a compound, concurrently converting the other oxygen atom to water. SO, VAT-1, and FOXRED 2 could promote the oxidation of protein and lipids. Pan et al. [93] also showed that protein oxidation was the primary factor associated with the quality deterioration of *Litopenaeus vannamei.*

## 4. Conclusions

The findings of this study demonstrated significant quality deterioration in both goat milk and cow milk during cold storage, such as nutrient loss, oxidative damage to proteins, and the disruption of their structural integrity. Goat milk exhibited a notably greater extent of lipid and protein oxidation compared to cow milk. This also resulted in significant changes to the protein structure of goat milk, including the increased exposure of free sulfhydryl groups and hydrophobic groups. These changes promoted hydrophobic interactions and facilitated the transformation of mercaptan groups. Therefore, the degree of protein aggregation in goat milk was higher during cold storage. This study provides a new insight into the quality changes in goat milk and cow milk during refrigeration, providing a theoretical basis for subsequent processing. However, the specific difference in oxidative sensitivity between goat milk and cow milk protein needs further investigation.

## Figures and Tables

**Table 1:** Changes in main components of raw goat milk and cow milk during cold storage.

Storage Time (Day)	Protein (%)		Fat (%)		Lactose (%)		SNF (%)		TS (%)	
	Goat Milk	Cow Milk	Goat Milk	Cow Milk	Goat Milk	Cow Milk	Goat Milk	Cow Milk	Goat Milk	Cow Milk
0	3.98 ± 0.01 ^a,X^	3.45 ± 0.00 ^A,Y^	3.58 ± 0.02 ^a,Y^	4.04 ± 0.01 ^A,X^	3.95 ± 0.01 ^a,Y^	3.96 ± 0.00 ^B,X^	8.17 ± 0.01 ^ab,X^	7.92 ± 0.01 ^A,Y^	11.61 ± 0.02 ^ab,Y^	12.00 ± 0.02 ^A,X^
1	3.94 ± 0.01 ^b,X^	3.44 ± 0.01 ^AB,Y^	3.55 ± 0.01 ^ab,Y^	3.99 ± 0.01 ^B,X^	3.96 ± 0.01 ^a,X^	3.98 ± 0.01 ^A,X^	8.19 ± 0.01 ^ab,X^	7.93 ± 0.01 ^A,Y^	11.61 ± 0.02 ^ab,Y^	12.00 ± 0.01 ^A,X^
2	3.93 ± 0.02 ^b,X^	3.43 ± 0.01 ^B,Y^	3.55 ± 0.02 ^b,Y^	3.94 ± 0.01 ^CD,X^	3.95 ± 0.01 ^a,Y^	3.98 ± 0.01 ^A,X^	8.15 ± 0.04 ^b,X^	7.93 ± 0.01 ^A,Y^	11.60 ± 0.03 ^ab,Y^	11.95 ± 0.02 ^B,X^
3	3.99 ± 0.01 ^a,X^	3.44 ± 0.02 ^AB,Y^	3.55 ± 0.01 ^b,Y^	3.98 ± 0.01 ^B,X^	3.92 ± 0.02 ^a,Y^	3.97 ± 0.01 ^AB,X^	8.19 ± 0.01 ^a,X^	7.96 ± 0.01 ^A,Y^	11.64 ± 0.03 ^a,Y^	12.00 ± 0.02 ^A,X^
5	3.95 ± 0.02 ^b,X^	3.44 ± 0.01 ^AB,Y^	3.52 ± 0.01 ^c,Y^	3.95 ± 0.01 ^C,X^	3.96 ± 0.01 ^a,X^	3.97 ± 0.01 ^AB,X^	8.21 ± 0.01 ^a,X^	7.96 ± 0.03 ^A,Y^	11.57 ± 0.04 ^bc,Y^	11.93 ± 0.04 ^B,X^
7	3.94 ± 0.02 ^b,X^	3.43 ± 0.02 ^B,Y^	3.49 ± 0.02 ^d,Y^	3.93 ± 0.02 ^D,X^	3.82 ± 0.06 ^a,Y^	3.93 ± 0.02 ^C,X^	8.11 ± 0.03 ^c,X^	7.87 ± 0.02 ^A,Y^	11.53 ± 0.04 ^c,Y^	11.91 ± 0.03 ^B,X^

Different letters (a–d and A–D) indicate significant differences (*p* < 0.05) between samples (lowercase letters for goat milk; capital letters for cow milk). Different letters (X, Y) indicate significant differences (*p* < 0.05) between samples of different types of milk stored for the same period.

**Table 2:** Changes in pH, acidity, total number of aerobic bacterial colonies, and protease activity of raw goat milk and cow milk during cold storage.

Storage Time (Day)	pH		Acidity (°T)		Total Colony (lgCFU/mL)		Protease Activity (△OD/h/mL)	
	Goat Milk	Cow Milk	Goat Milk	Cow Milk	Goat Milk	Cow Milk	Goat Milk	Cow Milk
0	6.701 ± 0.001 ^b,Y^	6.801 ± 0.001 ^A,X^	13.61 ± 0.40 ^e,X^	10.68 ± 0.72 ^E,Y^	4.00 ± 0.04 ^f,X^	4.04 ± 0.03 ^F,X^	0.117 ± 0.002 ^e,X^	0.037 ± 0.002 ^F,Y^
1	6.681 ± 0.001 ^c,Y^	6.802 ± 0.002 ^A,X^	14.61 ± 0.20 ^d,X^	11.64 ± 0.94 ^E,Y^	4.76 ± 0.17 ^e,X^	5.01 ± 0.02 ^E,X^	0.290 ± 0.002 ^d,X^	0.210 ± 0.001 ^E,Y^
2	6.710 ± 0.010 ^a,Y^	6.751 ± 0.001 ^B,X^	15.01 ± 0.41 ^cd,X^	13.06 ± 0.32 ^D,Y^	5.39 ± 0.05 ^d,X^	5.40 ± 0.08 ^D,X^	0.447 ± 0.007 ^c,X^	0.347 ± 0.003 ^D,X^
3	6.681 ± 0.001 ^c,X^	6.643 ± 0.002 ^C,Y^	15.23 ± 0.23 ^c,X^	14.57 ± 0.53 ^C,X^	6.12 ± 0.04 ^c,X^	6.02 ± 0.03 ^C,Y^	0.453 ± 0.005 ^c,X^	0.423 ± 0.003 ^C,X^
5	6.551 ± 0.001 ^d,X^	6.512 ± 0.003 ^D,Y^	17.91 ± 0.34 ^b,Y^	18.81 ± 0.16 ^B,X^	6.40 ± 0.03 ^b,X^	6.39 ± 0.04 ^B,X^	0.530 ± 0.003 ^b,X^	0.523 ± 0.002 ^B,X^
7	6.411 ± 0.002 ^e,X^	6.393 ± 0.002 ^E,Y^	19.60 ± 0.25 ^a,Y^	20.50 ± 0.23 ^A,X^	7.33 ± 0.06 ^a,X^	7.41 ± 0.05 ^A,X^	0.733 ± 0.002 ^a,X^	0.647 ± 0.001 ^A,Y^

Different letters (a–f and A–F) indicate significant differences (*p* < 0.05) between samples (lowercase letters for goat milk; capital letters for cow milk). Different letters (X, Y) indicate significant differences (*p* < 0.05) between samples of different types of milk stored for the same period.

## Data Availability

The original contributions presented in the study are included in the article. Further inquiries can be directed to the corresponding authors.
